# Peripapillary Vascular Reactivity in Primary Open-Angle Glaucoma With High Myopia by Using Optical Coherence Tomography Angiography

**DOI:** 10.3389/fmed.2022.850483

**Published:** 2022-03-18

**Authors:** Xintong Fan, Huan Xu, Ruyi Zhai, Qilian Sheng, Yanan Sun, Tingting Shao, Xiangmei Kong

**Affiliations:** ^1^Eye Institute and Department of Ophthalmology, Eye and ENT Hospital, Fudan University, Shanghai, China; ^2^NHC Key Laboratory of Myopia, Fudan University, Shanghai, China; ^3^Key Laboratory of Myopia, Chinese Academy of Medical Sciences, Shanghai, China; ^4^Shanghai Key Laboratory of Visual Impairment and Restoration, Shanghai, China

**Keywords:** optical coherence tomography angiography, primary open-angle glaucoma (POAG), high myopia (HM), hyperoxia, vasoreactivity, vascular response, retinal microcirculation

## Abstract

**Purpose:**

To evaluate peripapillary vascular reactivity in primary open-angle glaucoma (POAG) with and without high myopia (HM) by using optical coherence tomography angiography (OCTA).

**Methods:**

This prospective study enrolled 48 eyes with POAG, including 16 and 32 eyes with and without HM, respectively. The retinal peripapillary vessel density (VD) was repeatedly assessed using OCTA at baseline and after a hyperoxia test (breathing 80% oxygen). The VD changes between different oxygenation conditions were calculated to reflect the vasoreactivity. Linear regression was performed to determine the relationship between myopia and retinal vascular reactivity in patients with POAG. Systemic hemodynamic characteristics were also evaluated under both conditions.

**Results:**

The VD was significantly reduced after hyperoxia in the whole image (baseline and hyperoxia: 41.4 ± 4.5 and 38.8 ± 4.4, respectively, *P* < 0.001) and in the peripapillary regions (44.3 ± 5.7 and 41.1 ± 5.4, respectively, *P* < 0.001) in POAG eyes without HM. However, in eyes with HM, the whole-image VD in hyperoxia was not significantly different from the baseline (baseline and hyperoxia: 40.5 ± 6.2 and 40.2 ± 6.2, respectively, *P* = 0.481). The VD changes in eyes with HM were significantly smaller than those in eyes without HM in both the whole image (0.3 ± 1.8 and 2.6 ± 2.0, respectively, *P* < 0.001) and peripapillary regions (1.1 ± 2.0 and 3.2 ± 2.3, respectively, *P* = 0.003). Linear regression results showed a significant correlation between retinal vascular reactivity and spherical equivalent (SE) (β = 0.28, *P* < 0.001, *R*^2^ = 0.31) and axial length (AL) (β = −0.72, *P* < 0.001, *R*^2^ = 0.33).

**Conclusion:**

Retinal vasoreactivity of peripapillary capillaries in POAG eyes with HM was significantly impaired in comparison with that in POAG eyes without HM. A lower peripapillary vascular response was significantly associated with worse SE and elongated AL.

## Introduction

Primary open-angle glaucoma (POAG), as a blindness-causing disease, is characterized by optic nerve injury and progressive visual field (VF) loss (1, 2). Among several risk factors for POAG, myopia, especially high myopia (HM), is an established risk factor proved by population-based studies (3–5). Previous literature have revealed that the risk of POAG in HM patients is six times higher than in those without HM, and HM is highly correlated with the progression of glaucoma (6, 7). However, the underlying role and mechanism of HM in glaucoma pathogenesis has not been fully established.

Optical coherence tomography angiography (OCTA) allows high-resolution retinal microvasculature imaging in an non-invasive way (8), and its application has facilitated further investigation of the effect of myopia on ocular microcirculation in POAG. Previous OCTA reports have shown that vessel density (VD) is related to the severity of VF damage in glaucomatous eyes with HM (9, 10). Peripapillary microvessel evaluation has also shown distinct value in detecting glaucoma damage in HM (11, 12). However, the existing research on this topic has focused on microvascular structural changes, and retinal vascular functional changes was rarely explored in glaucomatous eyes with HM.

Vasoreactivity, which is also known as vascular autoregulation, is the term used to refer to the intrinsic ability of humans to maintain sufficient and stable blood flow by self-adjustment of the vascular system under various metabolic conditions (13). Analyses of vasoreactivity are indispensable in investigations of POAG pathophysiology. Vasculature in the cerebrum and retina has been reported to have an impaired reactivity in patients with POAG (14–16). Considering that ocular blood flow is playing a key part in developing glaucomatous eyes with HM, determination of the role of retinal vascular reactivity, which reflects vascular function, in the effects of myopic factors on POAG is important.

During hyperoxia condition, the retinal vessels were supposed to contract and decrease the perfusion in healthy subjects, which was reflected by the decrease of vessel density in OCTA measurements (17, 18). We had previously reported an easy, safe, and reproducible method using OCTA and a hyperoxia test to detect retinal microvascular reactivity in healthy subjects (18), as well as identify impaired vascular response in the optic nerve head (ONH) of POAG patients compared with healthy controls (19). Here we examined the different vasoreactivity in POAG eyes with and without HM, and further explored the potential relationship between myopia and ONH vascular autoregulation in POAG patients. Our study would contribute to a better understanding of glaucoma pathophysiology.

## Materials and Methods

### Ethics Statement

This prospective study followed the principles of the Declaration of Helsinki and was approved by the Institutional Review Board of the Eye and ENT Hospital of Fudan University, Shanghai, China (No. 2014043). Before enrolled in the study, all participants gave the informed consent and understood the nature of the study and its possible consequences.

### Participants

A total of 48 eyes of 48 POAG patients were recruited from the glaucoma clinic in the Eye and ENT Hospital of Fudan University between January 2021 and April 2021.

The diagnostic criteria for POAG were glaucomatous damages of optic nerve [including alterations of optic disk and thinning of retinal nerve fiber layer (RNFL)] and associated glaucomatous VF defects in at least two reliable VF tests, as well as open chamber angles in both eyes examined by gonioscopy. POAG diagnosis was confirmed by glaucoma specialist (XK). Participants included in the study were aged >18 years and had a best-corrected visual acuity (BCVA) of ≥20/40 (9, 10). Patients were excluded from the study if they had histories of intraocular surgery or other eye diseases (i.e., uveitis, severe cataracts, evident vitreous capacity, retinopathy, neuro-ophthalmic diseases) or loss of central fixation. In cases when both eyes could be included in the study, we randomly selected one eye. All participants had normal intraocular pressure (IOP) (<21 mmHg) on the test day with regular IOP-lowering eyedrop treatment. The included eyes were divided into two groups: POAG with and without HM. HM was defined as axial length (AL) higher than 26.0 mm or spherical equivalent (SE) less or equal to −6.0 diopters (D) (10).

Participants with diabetes, hypertension, other microvascular disorders, or a smoking history were excluded from the study, considering the potential effects of these conditions on retinal microcirculation and vasoreactivity (20, 21). Those with respiratory or cardiopathy diseases were also excluded because of the possible risks associated with inhalation of oxygen (22, 23).

### Ophthalmic Examinations and Study Protocol

All included participants underwent comprehensive ophthalmic examinations: slit-lamp microscopy and gonioscopy, Goldmann applanation tonometry for measurement of IOP, complete fundus examination, measurement of BCVA, refractive error, Lenstar examination of AL and central corneal thickness, and VF testing (Humphrey field analyzer, Carl Zeiss Meditec, Dublin, CA, United States). Results with ≤15% false-positive and false-negative rates and ≤20% fixation losses in VF tests were shown in all participants and were considered reliable. A spectral-domain optical coherence tomography system (RTuve XR Avanti; Optovue, Fremont, CA, United States) was used to evaluate retinal structure characteristics, including RNFL thickness, rim and disk area, as well as the ganglion cell complex (GCC) thickness. Only high-quality OCT images were included with a signal strength index of >60.

The detailed study protocol was previously described (18, 19). In brief, all participants were asked to avoid consuming alcohol or caffeine for at least 12 h before the test and were required to sit for 20 min under a room air environment before the hyperoxia provocation. Pulse rate (PR), blood pressure (BP), and ONH VD measured by OCTA were repeatedly evaluated and recorded at the baseline and after the hyperoxia test (breathing 80% oxygen at a flow rate of 15 L/min for 5 min) (24, 25), and the hyperoxia gas condition was maintained until all the second evaluations were completed. The mean arterial pressure (MAP) was calculated by the formula “diastolic BP + 1/3 (systolic BP - diastolic BP),” and the mean ocular perfusion pressure (MOPP) was calculated by “2/3 MAP – IOP.”

### Optical Coherence Tomography Angiography Imaging

**FIGURE 1:**
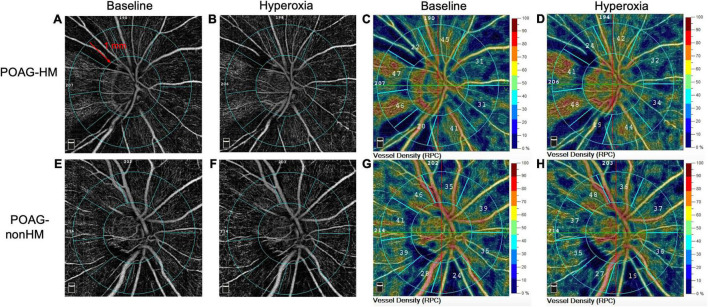
Representative optical coherence tomography angiography (OCTA) images of the capillary vessel density around the optic disk in a primary open-angle glaucoma (POAG) eye with high myopia (POAG-HM) **(A–D)** and a POAG eye without high myopia (POAG-nonHM) **(E–H)** at baseline and during hyperoxia. The range of the peripapillary region of the 1-mm-wide annulus is defined in panel **(A)**. RPC, radial peripapillary capillary.

All OCTA scans were obtained from a commercial spectral-domain OCTA device (RTuve XR Avanti; Optovue, Fremont, CA, United States) with the split-spectrum amplitude-decorrelation angiography (SSADA) algorithm (26). A total of 216 A-scans formed into each B-scan, and B-scans were merged into three-dimensional OCTA scans by the device. After acquiring the scans, motion artifacts were automatically removed. Scanning of a 4.5 mm × 4.5 mm region centered on ONH was conducted repeatedly at baseline and after 5 min of oxygen breathing. As shown in Figure 1A, a 1-mm wide annulus outside the boundary of the ONH was defined as the peripapillary region. The custom software automatically analyzed radial peripapillary capillary (RPC) from the inner limiting membrane to RNFL. An en-face retinal angiogram was produced, and the software automatically masked the large vessels and only calculated the capillary vessel density. To improve the image quality and reduce residual motion artifacts, the eye-tracking function was employed during scanning. For analyses, we only included images with a high-quality signal strength index >7/10 with no motion artifacts.

### Statistical Analysis

Data are expressed as mean ± standard deviation. The differences in continuous data between POAG eyes with and without HM were evaluated by Student’s *t-test* (for normally distributed data) and Mann–Whitney U test (for non-normally distributed data). Comparisons of the blood flow variables and OCTA metrics between baseline and hyperoxia conditions were assessed using Student’s paired *t*-tests. Categorical data were assessed by Fisher’s exact test. Univariate and multivariate linear regressions were applied to determine associations between ONH vasoreactivity (VD difference between baseline and hyperoxia) and relevant variables. Statistical analysis was performed by STATA ver. 15.1 (College Station, TX, United States). Between-group statistical differences were regarded as significant at *P* < 0.05.

## Results

### Baseline and Demographic Data

The baseline data were comparable between glaucomatous eyes with and without HM in terms of sex, laterality, PR, disease duration, antiglaucoma medications, IOP, BCVA, central corneal thickness, RNFL thickness, rim area, GCC thickness, focal loss volume, percentage of global loss volume, VF, and whole-image and peripapillary VD (*P* = 0.168–1.000) (Table 1). The two groups had significantly different SE and AL (all *P* < 0.001), and the disk area was lower in POAG eyes with HM than those without (*P* = 0.027). However, age and baseline blood flow variables including systolic and diastolic arterial pressure (SAP and DAP), MAP, and MOPP, were not comparable between the two groups (*P* = 0.002–0.024). Subsequently, we conducted linear regression to determine whether these variables were associated with ONH vascular responses, the main outcome we focused on in this study (Supplementary Table 1). Univariate regression results indicated that the SAP, DAP, MAP, and MOPP showed no correlation with RPC-VD differences (*P* = 0.055–0.755). Although age showed a significant correlation with VD difference in univariate analysis, in multivariate models that included the axial length variable, the VD differences were significantly correlated with axial length (*P* < 0.001–0.004), but not with age (*P* = 0.822–0.970). Therefore, the differential age and hemodynamic variables between the two groups did not influence our exploration of the relationship between HM and ONH vasoreactivity in POAG patients.

**TABLE 1:** Baseline and demographic data.

	Eyes with high	Eyes without high	*P-*value
	myopia, *n* = 16	myopia, *n* = 32	
Age, years	32.9 ± 7.0	43.9 ± 12.7	0.002
Male/Female, n	9/7	18/14	1.000
Right/Left, n	10/6	16/16	0.542
SAP, mm Hg	114.4 ± 15.1	126.3 ± 15.3	0.016
DAP, mm Hg	75.9 ± 11.9	82.8 ± 8.0	0.024
MAP, mm Hg	88.8 ± 12.8	97.3 ± 9.9	0.016
MOPP, mm Hg	43.2 ± 8.0	49.4 ± 7.3	0.011
PR, bpm	75.9 ± 10.5	77.1 ± 10.8	0.737
Disease duration, months	24.9 ± 18.4	19.8 ± 24.1	0.480
Anti-glaucoma eyedrops, n	1.75 ± 0.58	1.47 ± 0.88	0.253
IOP, mm Hg	16.0 ± 4.3	15.4 ± 2.7	0.584
BCVA, logMAR	0.07 ± 0.10	0.04 ± 0.09	0.349
SE, diopter	−9.0 ± 2.6	−1.7 ± 2.6	<0.001
CCT, μm	525.9 ± 38.5	538.7 ± 30.4	0.269
AL, mm	27.6 ± 0.8	24.4 ± 1.0	<0.001
RNFL thickness, μm	75.8 ± 12.8	78.0 ± 10.2	0.512
Rim area, mm^2^	0.67 ± 0.25	0.77 ± 0.26	0.217
Disk area, mm^2^	1.99 ± 0.38	2.27 ± 0.41	0.027
GCC thickness, μm	79.4 ± 12.5	80.0 ± 10.8	0.852
FLV%	5.42 ± 3.68	7.12 ± 4.06	0.168
GLV%	17.5 ± 11.5	16.9 ± 9.9	0.868
Visual field, MD, dB	−7.3 ± 9.0	−6.1 ± 5.5	0.575
**Vessel density**			
Whole image	40.5 ± 6.2	41.4 ± 4.5	0.574
Peripapillary	42.1 ± 7.6	44.3 ± 5.7	0.261

*Data are expressed as mean ± standard deviation. SAP, systolic arterial pressure; DAP, diastolic arterial pressure; MAP, mean arterial pressure; MOPP, mean ocular perfusion pressure; PR, pulse rate; IOP, intraocular pressure; BCVA, best-corrected visual acuity; SE, spherical equivalent; CCT, central corneal thickness; AL, axial length; RNFL, retinal nerve fiber layer; GCC, ganglion cell complex; FLV%, focal loss volume percentage; GLV%, global loss volume percentage; MD, mean deviation; dB, decibel.*

### Systemic Reactivity in Primary Open-Angle Glaucoma Eyes With and Without High Myopia

No significant differences between baseline and hyperoxic conditions were detected in systemic BP and MOPP in POAG eyes with and without HM (*P* = 0.074–0.944) (Table 2). The PR was significantly decreased after hyperoxia in comparison with baseline in both groups (*P* < 0.001, *P* = 0.025, respectively) (Table 2). Moreover, since the PR decreased more in HM than in non-HM group, we also performed linear regression and detected no association between PR and retinal vasoreactivity (*P* = 0.289–0.472).

**TABLE 2:** Blood flow variables at baseline and after hyperoxia.

	Eyes with high	*P*-value	Eyes without high	*P*-value
	myopia, *n* = 16		myopia, *n* = 32	
**SAP, mm Hg**				
Baseline	114.4 ± 15.1	–	126.3 ± 15.3	–
Hyperoxia	115.8 ± 13.9	0.164	128.4 ± 13.3	0.944
**DAP, mm Hg**				
Baseline	75.9 ± 11.9	–	82.8 ± 8.0	–
Hyperoxia	76.2 ± 13.7	0.269	84.3 ± 6.5	0.141
**MAP, mm Hg**				
Baseline	88.8 ± 12.8	–	97.3 ± 9.9	–
Hyperoxia	89.4 ± 13.6	0.074	99.1 ± 8.4	0.388
**MOPP, mm Hg**				
Baseline	43.2 ± 8.0	–	49.4 ± 7.3	–
Hyperoxia	44.3 ± 8.6	0.074	50.5 ± 6.8	0.388
**PR, bpm**				
Baseline	75.9 ± 10.5	–	77.1 ± 10.8	–
Hyperoxia	66.9 ± 7.7	<0.001	74.8 ± 11.7	0.025

*Data are expressed as mean ± standard deviation.*

*SAP, systolic arterial pressure; DAP, diastolic arterial pressure; MAP, mean arterial pressure; MOPP, mean ocular perfusion pressure; PR, pulse rate.*

### Retinal Vascular Reactivity in Primary Open-Angle Glaucoma Eyes With and Without High Myopia

The VD of RPC decreased significantly after hyperoxia test in comparison with the baseline in non-HM eyes in both the whole image and the peripapillary regions (all *P* < 0.001) (Table 3). In POAG eyes with HM, the RPC-VD after hyperoxia provocation was similar to baseline in the whole image (*P* = 0.481), but was reduced in the peripapillary region in comparison with the baseline (*P* = 0.040) (Table 3). The absolute and relative differences in RPC-VD comparing hyperoxia conditions to baseline were significantly smaller in POAG eyes with HM than those in eyes without HM in both the whole image (all *P* < 0.001) and peripapillary regions (*P* = 0.003, *P* = 0.004, respectively) (Table 4). Figure 1 presents examples of OCTA images in POAG eyes with and without HM under the two gas conditions.

**TABLE 3:** Retinal vessel density at baseline and after hyperoxia in primary open-angle glaucoma (POAG) eyes with and without high myopia.

Vessel density	Eyes with high	*P*-value	Eyes without high	*P*-value
	myopia, *n* = 16		myopia, *n* = 32	
**Whole image**				
Baseline	40.5 ± 6.2	–	41.4 ± 4.5	–
Hyperoxia	40.2 ± 6.2	0.481	38.8 ± 4.4	<0.001
**Peripapillary**				
Baseline	42.1 ± 7.6	–	44.3 ± 5.7	–
Hyperoxia	41.0 ± 7.4	0.040	41.1 ± 5.4	<0.001

*Data are expressed as mean ± standard deviation.*

**TABLE 4:** Retinal vascular response to hyperoxia in POAG eyes with and without high myopia.

Vessel density	Eyes with high	Eyes without high	*P*-value
	myopia, *n* = 16	myopia, *n* = 32	
Absolute difference*			
Whole image	0.3 ± 1.8	2.6 ± 2.0	<0.001
Peripapillary	1.1 ± 2.0	3.2 ± 2.3	0.003
**Relative difference^†^**			
Whole image	0.7% ± 5.2%	6.3% ± 4.7%	<0.001
Peripapillary	2.5% ± 5.3%	7.2% ± 4.9%	0.004

*Data are expressed as mean ± standard deviation.*

*Absolute difference: Value of Baseline minus hyperoxia; ^†^Relative difference: Ratio of absolute difference and baseline value (%).

### Relationships Between Retinal Vascular Reactivity and Spherical Equivalent and Axial Length in Primary Open-Angle Glaucoma Eyes With and Without High Myopia

**FIGURE 2:**
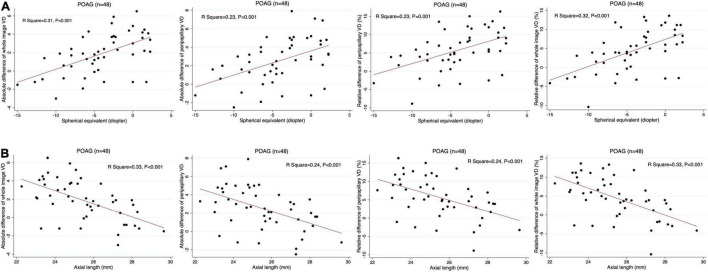
Scatter plots presenting the linear correlations between vessel density (VD) differences and spherical equivalent and axial length in POAG eyes. Absolute and relative difference of the whole-image and peripapillary VD are significantly correlated with spherical equivalent **(A)** and axial length **(B)** in POAG eyes, respectively.

In linear regression analysis of all POAG patients (*n* = 48), smaller RPC-VD differences, including those for the whole image and the peripapillary areas, were significantly correlated with higher myopic SE and longer AL in patients with POAG (*P* ≤ 0.001) (Table 5). In multivariate models that included age variables, the associations between ONH vasoreactivity and SE and AL were consistent with the results of univariate analysis (*P* = 0.001–0.007) (Table 5). Moreover, age showed no correlation with ONH vascular reactivity in the multivariate models (*P* = *0.822*–0.990). The linear relationships between the ONH vascular response and SE and AL in POAG patients are displayed in Figure 2.

**TABLE 5:** Linear relationships between retinal vasoreactivity and spherical equivalent and axial length in POAG.

	Correlation Variables	Univariate			Multivariate		
		β (95% CI)	*P-*Value	*R* ^2^	β (95% CI)	*P-*Value	*R* ^2^
Wi-AD	SE	0.28 (0.16–0.41)	<0.001	0.31	0.27 (0.11–0.44)	0.001	0.31
	Axial length	−0.72 (−1.02 to −0.41)	<0.001	0.33	−0.69 (−1.07 to −0.31)	0.001	0.33
Peri-AD	SE	0.26 (0.12–0.40)	0.001	0.23	0.26 (0.08–0.45)	0.007	0.23
	Axial length	−0.66 (−1.00 to −0.31)	<0.001	0.24	−0.65 (−1.09 to −0.21)	0.004	0.24
Wi-RD	SE	0.71 (0.40–1.02)	<0.001	0.32	0.70 (0.30–1.10)	0.001	0.32
	Axial length	−1.79 (−2.53 to −1.04)	<0.001	0.33	−1.73 (−2.68 to −0.78)	0.001	0.33
Peri-RD	SE	0.61 (0.28–0.94)	0.001	0.23	0.61 (0.18–1.03)	0.006	0.23
	Axial length	−1.52 (−2.32 to −0.73)	<0.001	0.24	−1.50 (−2.51 to −0.50)	0.004	0.24

*P-values are obtained with linear regression. Multivariate linear models are adjusted for age.*

*CI, confidence interval; SE, spherical equivalent; Wi, whole image; Peri, peripapillary; AD, absolute difference; RD, relative difference.*

## Discussion

In the study, we investigated retinal vasoreactivity in POAG eyes with and without HM, and determined the relationship between myopia and ONH vascular reactivity in glaucomatous eyes by using OCTA and hyperoxia tests. We found that POAG eyes with HM presented significantly impaired vascular autoregulation than eyes without HM. Moreover, we observed a strong significant linear correlation between ONH vasoreactivity and SE and AL in patients with POAG.

In our study, POAG patients with HM were younger than patients without HM. Moreover, probably due to the age difference between the groups, the BP of patients without HM was significantly higher than that of patients with HM, although patients with hypertension were excluded from the study. To confirm that the ONH vasoreactivity difference between the two groups was caused by HM and not by the differences in age and systemic blood flow variables, we performed linear regression to test the effects of these factors on the ONH vascular response. We found that none of the hemodynamic variables were associated with vascular response, and age showed no correlation with vasoreactivity in multivariate models (Supplementary Table 1). In addition, the regression models were also adjusted for age when investigating the relationship between myopia and vasoreactivity, and we found that the ONH vascular response was still significantly correlated with SE and AL after adjustment for age (Table 5). Therefore, the confounding effect of age and blood flow factors did not influence our findings on HM and vascular reactivity in glaucomatous eyes.

Peripapillary evaluations in glaucomatous eyes with HM are challenging due to structural variations such as a tilted disk and peripapillary atrophy (27). In OCT examinations, segmentation error is the main concern in HM eyes due to AL elongation and large peripapillary atrophy (28, 29). Segmentation errors in OCT may prejudice the precise assessment of peripapillary RNFL thickness and cause inaccurate glaucoma diagnosis (30, 31). In this respect, OCTA presents a unique advantage in peripapillary evaluation of HM eyes, since the visualization of retinal VD does not demand strictly accurate segmentation as much as the measurement of thickness does; consequently, it is less affected by HM-related anatomic variation (10). Previous studies indicated that peripapillary VD had stronger association with VF sensitivity than RNFL thickness did in glaucomatous eyes with HM, revealing that OCTA may be more useful in monitoring visual functional changes than OCT in HM eyes. However, these studies all focused on retinal vascular structure characteristics, and there have been no studies investigating vascular function alterations in high myopic glaucoma.

Vasoreactivity, which reflects the function of the vasculature, is an extremely critical aspect in research on POAG. Previous studies have used dynamic vessel analysis and laser Doppler flowmetry to detect decreased vasoreactivity in retinal vessels and cerebral vessels in patients with POAG (14, 32, 33). Our recent study also showed that ONH vascular reactivity was significantly impaired in eyes with POAG using OCTA (19). Furthermore, in the current study, we observed that POAG eyes with HM had worse vasoreactivity than eyes without HM. In POAG eyes without HM, RPC-VD still significantly decreased in response to hyperoxia provocation, which was consistent with our previous findings (19). However, in eyes with HM, the RPC-VD was not significantly reduced after hyperoxia in the whole image, and the VD differences between baseline and hyperoxia conditions were markedly lower than those in eyes without HM. These results imply that impaired ONH vascular reactivity may be involved in the pathophysiology and development of high myopic glaucoma, and retinal vascular dysfunction may aggravate the progression of POAG in eyes with HM.

We further examined the associations between refractive errors and ONH vascular response in patients with POAG. Linear regression was performed in all 48 patients with POAG, both with and without HM. Interestingly, we found that a lower ONH vasoreactivity was significantly correlated with a worse SE and elongated AL in both univariate and multivariate models after adjustment for age. This result indicates that refractive error may be an independent influencing factor for vascular reactivity in POAG. In accordance with our results, Lin et al. found that retinal VD decreased faster in POAG eyes with HM than in those without during longitudinal follow-up (34). Additionally, Li et al. reported a negative correlation between retinal VD and AL in myopic eyes, which is also consistent with our findings for retinal vasoreactivity (35). The mechanism underlying the effect of myopia on retinal vasoreactivity may result from the stretching of the microvascular network during AL elongation (35). This structural alteration may subsequently lead to or accompany microvascular dysfunction in glaucomatous eyes with HM. Therefore, AL elongation in myopic eyes resulted in not only vascular structural changes (decreased VD), but also functional changes in vascular autoregulation (impaired vasoreactivity), which should also be highlighted during the follow-up of high myopic glaucoma.

Our study also had certain limitations. First, the age and blood flow variables were significantly heterogenous between POAG eyes with and without HM, which may have affected our study outcomes. However, our findings proved that there was no correlation between these variables and ONH vascular reactivity, and we also made the adjustment when detecting the relationship between HM and vasoreactivity. Therefore, we believe that the confounding effect of the baseline factors was negligible in our study. Second, we did not exclude eyes using IOP-lowering medications, and some antiglaucoma agents may affect ocular microcirculation and vascular reactivity. However, recruitment of sufficient POAG patients while excluding those using any IOP-lowering eyedrops is difficult in clinical practice, and the number of eyes receiving antiglaucoma medication was comparable between the groups with and without HM in our study. Therefore, the application of these agents did not significantly affect our main findings. Third, to ensure data reliability, we only included high-quality OCTA images in the analysis of our study, which may limit the availability of the results in eyes with severe visual loss and poor-quality OCTA scans. Forth, we only included POAG eyes with and without HM, to evaluate retinal vasoreactivity in eyes with HM but without POAG is also important, and is needed in the future investigation. Finally, our sample size was relatively limited. Larger and longitudinal studies including more participants of various ages and ethnic backgrounds would provide more insights into ocular microcirculation in glaucomatous eyes with HM.

In summary, our study explored optic disk vascular reactivity in eyes with POAG and HM. In comparison with glaucomatous eyes without HM, those with HM showed significantly impaired vasoreactivity, and this impairment was significantly associated with worse SE and longer AL. Thus, our study revealed alterations in retinal vascular autoregulation in POAG eyes with HM, which may help improve our understanding of myopic glaucoma pathophysiology and contribute to research about HM in POAG development and progression. The feasibility and usefulness of OCTA in capillary functional analysis have also been highlighted and expanded in our study.

## Data Availability Statement

The raw data supporting the conclusions of this article will be made available by the authors, without undue reservation.

## Ethics Statement

The studies involving human participants were reviewed and approved by the Institutional Review Board of the Eye and ENT Hospital of Fudan University, Shanghai, China (No. 2014043). The patients/participants provided their written informed consent to participate in this study.

## Author Contributions

XF designed the study, collected the data, performed the data analysis and data interpretation, and wrote the manuscript. HX, RZ, QS, and YS contributed to the data discussion. XK and TS contributed to the data discussion and reviewed the manuscript. All authors contributed to the manuscript revision, read, and approved the submitted version.

## Conflict of Interest

The authors declare that the research was conducted in the absence of any commercial or financial relationships that could be construed as a potential conflict of interest.

## Publisher’s Note

All claims expressed in this article are solely those of the authors and do not necessarily represent those of their affiliated organizations, or those of the publisher, the editors and the reviewers. Any product that may be evaluated in this article, or claim that may be made by its manufacturer, is not guaranteed or endorsed by the publisher.

## Abbreviations

POAG: primary open-angle glaucoma
VF: visual field
OCTA: optical coherence tomography angiography
VD: vessel density
ONH: optic nerve head
RNFL: retinal nerve fiber layer
BCVA: best-corrected visual acuity
IOP: intraocular pressure
AL: axial length
SE: spherical equivalent
GCC: ganglion cell complex
BP: blood pressure
MAP: mean arterial pressure
MOPP: mean ocular perfusion pressure
RPC: radial peripapillary capillary
SAP: systolic arterial pressure
DAP: diastolic arterial pressure.
