# Supplementary material for: Peripapillary Vascular Reactivity in Primary Open-Angle Glaucoma With High Myopia by Using Optical Coherence Tomography Angiography
Source: Front Med (Lausanne). 2022 Mar 18;9:850483. doi: 10.3389/fmed.2022.850483 (PMC8971362; doi:10.3389/fmed.2022.850483)
Supplement: Supplementary file 1 [file Table_1.pdf]

Supplementary Material

Supplementary Table S1. Linear regression results of correlations between the ONH vasoreactivity and baseline variables that were significantly different in the two study groups

		Univariate			Multivariate	
	Variable	β	*P* Value	R^2	β	*P* Value
Absolute Δ*						
Whole image	Age	0.07	0.010	0.13	0.006	0.822
	SAP	0.04	0.055	0.08	0.008	0.663
	DAP	0.04	0.201	0.04	-0.001	0.693
	MAP	0.05	0.104	0.06	-0.001	0.983
	MOPP	0.06	0.109	0.06	0.003	0.929
Peripapillary	Age	0.06	0.039	0.09	0.001	0.970
	SAP	0.02	0.252	0.03	-0.004	0.855
	DAP	0.01	0.755	0.002	-0.04	0.220
	MAP	0.02	0.478	0.01	-0.02	0.430
	MOPP	0.03	0.439	0.01	-0.03	0.556
Relative Δ*						
Whole image	Age	0.16	0.010	0.13	0.01	0.845
	SAP	0.09	0.056	0.08	0.02	0.678
	DAP	0.11	0.178	0.04	-0.02	0.750
	MAP	0.12	0.097	0.06	0.001	0.991
	MOPP	0.15	0.145	0.05	-0.009	0.921
Peripapillary	Age	0.14	0.036	0.09	0.005	0.945
	SAP	0.06	0.267	0.03	-0.01	0.820
	DAP	0.03	0.709	0.003	-0.09	0.249
	MAP	0.05	0.466	0.01	-0.05	0.443
	MOPP	0.06	0.556	0.008	-0.08	0.414

Multivariate models involve in each variable and axial length.

SAP, systolic arterial pressure; DAP, diastolic arterial pressure; MAP, mean arterial pressure; MOPP, mean ocular perfusion pressure

*Δ: Vessel density difference between baseline and hyperoxia
